# FoxM1 Directs STAT3 Expression Essential for Human Endometrial Stromal Decidualization

**DOI:** 10.1038/srep13735

**Published:** 2015-09-03

**Authors:** Yaling Jiang, Yixin Liao, Hui He, Qiliang Xin, Zhaowei Tu, Shuangbo Kong, Tongtong Cui, Bingyan Wang, Song Quan, Bing Li, Shuang Zhang, Haibin Wang

**Affiliations:** 1 Reproductive Medical Center, Department of Obstetrics and Gynecology, The Third Affiliated Hospital of Guangzhou Medical University, Guangzhou 510150, China; 2 State Key Laboratory of Reproductive Biology, Institute of Zoology, Chinese Academy of Sciences, Beijing 100101, China; 3 Center for Reproductive Medicine, Department of Obstetrics and Gynecology, Nanfang Hospital, Southern Medical University, Guangzhou 510515, China

## Abstract

Human endometrium decidualization, which involves endometrial stromal proliferation and differentiation, is a prerequisite for embryo implantation, thus successful pregnancy. The Forkhead Box M1 (FoxM1), previously known as HNF-3, HFH-11, MPP2, Win, and Trident, is a transcriptional factor that plays crucial roles in cell proliferation and cell cycle progression. However, the molecular mechanism of FoxM1 during human endometrial decidualization remains unexplored. In this study, we first found FoxM1 is dynamically expressed in human endometrium during menstrual cycle. Employing a human endometrial stromal cell (HESC) line, we then demonstrated that FoxM1 inhibition downregulates cyclin B1 expression, delaying G2/M phase transition during HESC proliferation. Additionally, loss of FoxM1 expression blocks the differentiation of HESCs in response to estrogen, progesterone, and dbcAMP. Applying chromatin immunoprecipitation (ChIP) technique and luciferase assay, we further approved that FoxM1 can transcriptionally active signal transducer and activator of transcription 3 (STAT3), ensuring normal HESC differentiation. Besides enriching our knowledge on molecular basis underlying stromal decidualization, these findings help to shed light on the potential molecular causes for the endometrial disorders in humans.

The human endometrium is a highly dynamic tissue that undergoes repeated cycles of proliferation, differentiation and regeneration driven by fluctuation of the estrogen (E_2_) and progesterone (P_4_) every 28 days^1^. Prior to ovulation, the uterus is at an E_2_-dominated proliferative phase (D1-14). Accompanied by occurrence of ovulation, the endometrium then enters a P_4_-dominated secretory phase (D15-28) during which the increase of P_4_ level induces profound remodeling and differentiation of the E_2_-primed stromal cells into larger, rounded decidual cells. This process, known as decidualization, comprises characteristically morphogenetic, biochemical and vascular changes, and is an essential prerequisite for blastocyst implantation and maintenance of the pregnancy^2^. The decidualized endometrium is emerging as an active gatekeeper to implantation in the human^3^. Abnormal decidualization, as a result of poor hormone responsiveness and/or impaired endometrial stromal cell growth, is often associated with a variety of endometrial and pregnancy complications, including unexplained infertility, recurrent spontaneous abortion, intrauterine growth retardation, preeclampsia, premature birth and other clinical gynecological diseases, such as endometriosis and endometrial cancer^4^,^5^. However, the underlying molecular basis that governing the process of endometrial decidualization remained poorly understood.

Employing transgenic mouse models and *in vitro* stromal cell lines, accumulating evidence has demonstrated that ovarian hormone-regulated endometrial decidualization is accompanied with dramatic transcriptional reprogramming. A wide range of transcription factor like PR, CCAAT/enhancer-binding protein β (C/EBPβ), HOXA10/11, FoxO1, and Klf12 have been identified successively as key regulators involved in the regulation of stromal cell decidualization through affecting the expression of decidual marker genes, such as prolactin (PRL) and insulin-like growth factor binding protein-1 (IGFBP-1)^6^,^7^,^8^,^9^,^10^. Moreover, these transcriptional factors function coordinately to form a cascade network during endometrial decidualization. For example, C/EBPβ mediates the biological functions of P_4_-PR signaling during decidualization^11^,^12^, while the signal transducer and activator of transcription 3 (STAT3) can function as a direct regulatory target of C/EBPβ involved in human endometrial decidualization^13^. However, it remained largely unexplored regarding the potential transcriptional regulatory network during endometrial proliferation and differentiation.

The Forkhead Box M1 (FoxM1), previously known as HNF-3, HFH-11, MPP2, Win, and Trident, is a transcriptional factor that plays a crucial role in cell proliferation, differentiation and transformation^14^. For example, FoxM1 can regulate a number of cell cycle key regulators, directing the cell cycle progression from G1 to S phase and at G2/M transition, as well as cytokinesis^15^,^16^. Loss of FoxM1 induces mitotic catastrophe and cell death due to mitotic spindle defects^17^,^18^. Moreover, aberrant expression of FoxM1 via influencing the cell cycle progress and metasis is often associated with various types of human malignancies^16^,^17^,^19^,^20^,^21^. Since the human endometrial cycle is characterized as a process involving dynamic cell cycle progression and tissue remolding, it was interesting to question whether the FoxM1 is an essential player during human endometrial stromal proliferation and decidual transformation.

In the present study, we revealed that FoxM1 is dynamically expressed in human menstrual endometrium. Employing the HESC line, we further demonstrated while FoxM1 is required for stromal cell proliferation by promoting G2/M progression, it acts as a critical player in regulating endometrial differentiation via directly targeting the expression of STAT3.

## Materials and Methods

### Human endometrial tissue samples

This study was approved by the Medical Ethics Committee of The Third Affiliated Hospital of Guangzhou Medical University and all works were performed in accordance with the Declaration of Helsinki. Informed consents were obtained for all the subjects before their inclusion in the study. Endometrial tissue subjects were collected from women who visited the Reproductive Medical Center, The Third Affiliated Hospital of Guangzhou Medical University from January to May 2013. The control subjects were selected from women who visited the clinic for the IVF procedure and the causes of marital infertility were fallopian tube obstruction or male azoospermia. None of subjects had received any hormonal treatment during the preceding three months. Seventy normal subjects with regular cycles (ranging from 25 to 35 days) were undergone endometrial sampling on a specific cycle day under a protocol approved by the Third Affiliated Hospital of Guangzhou Medical University. Proliferative phase samples were timed based on the patient’s cycle day, and luteal phase samples were timed using the subject’s urinary luteinizing hormone (LH) surge. Samples were categorized as either early proliferative (n = 12), late proliferative (n = 15), early secretory (n = 14), mid-secretory (n = 15), or late secretory (n = 14). The portions designated for RNA analysis were immediately placed in liquid nitrogen. The formalin-fixed portions were embedded in paraffin, and sectioned for immunohistochemistry. The histological sections were reviewed by two expert pathologists to verify the histological variants. All 70 samples were used for quantitative real-time PCR. Ten samples at each stage were used for immunohistochemistry, and all these slides were used to conduct semiquantitative histologic scoring (HSCORE) analysis.

### HESC culture, synchronization, and *in vitro* decidualization

Immortalized HESC line was purchased from the American Type Culture Collection (ATCC^R^ CRL-4003^TM^) and cultured according to the manufacturer’s instructions^22^. Briefly, HESCs were cultured in DMEM/F12 (Sigma) supplemented with 10% charcoal-stripped FBS (CS-FBS, Biological Industries) at 37 °C in a humidified chamber with 5% CO_2_. Synchronization of HESCs in the G0/G1 phase was achieved by serum starvation overnight as described previously^23^. To induce decidualization *in vitro*, HESCs were treated with differential medium (DMEM/F12 with 2% CS-FBS) containing 10 nM E2 (Sigma), 1 μM Medroxyprogesterone 17-acetate (MPA, Sigma), and 0.5 mM dibutyryl cAMP (dbcAMP, Sigma). The medium was changed every 48 h. Thiostrepton (Enzo) and pyridone 6 (Merck Millipore) were used to inhibit FoxM1 and the Janus kinase-STAT3 pathway in culture, respectively.

### Immunostaining

Endometrial tissues from three RIF subjects and fifteen various staged controls were examined. Immunostaining was performed as described previously^24^. The endometrial tissues were fixed in 4% paraformaldehyde (Sigma) and then embedded in paraffin. After deparaffinization, rehydration, antigen retrieval, inhibition of endogenous peroxidase activity, and blocking, sections were incubated with anti-FoxM1 antibody (1:500, Santa Cruz) overnight. HESCs cultured in chamber slides were fixed in 4% paraformaldehyde followed by permeabilization and blocking. Incubations with the respective primary antibodies including anti-FoxM1 (1:200, Santa Cruz), anti-cyclin B1 (1:600, Abcam) and anti-phospho-histone 3 (pH3, 1:200, CST) were performed overnight at 4°C. Fluorescence (cyanine 3)-conjugated secondary antibodies were used respectively to visualize the signal and nuclei were stained with DAPI (1 μg/ml, Sigma).

### RNA extraction and quantitative polymerase chain reaction (q-PCR)

Total RNA was extracted from tissue samples and cultured HESCs using the TRIzol RNA purification kit (Invitrogen), and 3 μg of total RNA was used to synthesized cDNA according to the manufacturer’s instructions. Expression levels of different genes were validated by q-PCR using SYBR Green Supermix (CWBio). All primers for q-PCR were listed in Supplemental Table 1. The mRNA expression level at any given time point or under a given condition was quantified as fold change (mean ± SEM) relative to the control condition after normalization with respect to the internal control GAPDH. All the experiments were repeated at least three times.

### Western blot

Western blotting analysis was performed as described previously^23^. Total protein extracts from cultured HESCs were separated on 10% SDS-PAGE gels. The primary antibodies were applied according to the provided recommendations: anti-FoxM1 (1:1000, ABclonal), anti-cyclin B1 (1:1000, Abcam), anti-pH3 (1:1000, CST), anti-cleaved caspase 3 (1:1000, ABclonal), anti-caspase 3 (1:1000, ABclonal), anti-caspase 9 (1:1000, ABclonal), anti-cleaved PARP (1:1000, Bioworld), anti-STAT3 (1:1000, CST), anti-phospho-STAT3 (1:1000, CST), anti-C/EBPβ (1:1000, Santa Cruz), respectively. Anti-β-actin (1:5000, Sigma) was used as the internal control. Bands were visualized using Thermo Supersignal West Pico Chemiluminescent substrate according to the manufacturer’s instructions. The intensity of bands was determined by using Quantity One software, and the quantitative analyses of gray-scale value of each target protein vs that of individual β-actin were performed.

### Cell proliferation assays

Cell proliferation viability was evaluated by MTS reagent (Promega) according to the manufacturer’s directions. HESCs were seeded on 96-well plates (5 × 10^3^/well), and cell numbers were analyzed at D0, D1, D2, D3 and D4 after addition of MTS. The experiments were repeated three times.

### Flow Cytometry

Flow cytometric analysis was performed as previously described^23^. HESCs were trypsinized, collected and fixed in 90% ethanol at different time-points after Synchronization. The fixed cells were incubated with 30 μg/ml propidium iodide (PI) and 300 μg/ml RNase A for 20 min before flow cytometry analysis.

### Chromatin immunoprecipitation (ChIP)

ChIP assay was performed as previously described^24^. HESCs were treated with E_2_+MPA+dbcAMP for 3 days to induce decidualization. Thereafter, HESCs (1 × 10^7^) were cross-linked, collected, lysed, then sheared by sonication until the average length of DNA was ∼500 bp as evaluated by agarose gel electrophoresis. 1% of the chromatin fragments were stored at −20 °C to be used later for non-precipitated total chromatin (input) for normalization. The rest of chromatin fragments were equally divided, and incubated overnight with 4 μg anti-FoxM1 antibody or 4 μg anti-rabbit IgG, as a negative control for nonspecific immunoprecipitation. The chromatin-antibody complex was incubated with protein A beads for 4 h, then the beads were washed repeatedly. The beads were suspended in elution buffer and the precipitated protein/DNA complexes were eluted from the antibodies/beads. The cross-linking was reversed, and then proteins were digested using proteinase K. Purified DNA served as the template for q-PCR using various primer sets to amplify specific regions of the STAT3 promoter.

### Luciferase reporter assay

HEK293T cells were cultured in DMEM (Hyclone) supplemented with 10% FBS (Hyclone) and applied to luciferase reporter assay. Luciferase reporter assay was carried out using the Dual-Luciferase Reporter Assay System (PGL3-Basic vector, Promega). The two fragments (F1: −755 ∼−1834; and F2: +9325 ∼ +9580) of the STAT3 promoter containing the predicted binding sites for FoxM1 were inserted into PGL3-Basic vector, respectively. A pFLAG-CMV vector expressing FoxM1 was constructed by cloning the full-length PCR product of human FoxM1. All the constructed plasmids were verified by DNA sequencing. After transfection, luciferase activity was detected and normalized to Renilla activity. The experiments were performed in triplicates and each experiment was repeated three times.

### Statistical analysis

SPSS 17.0 software was used for statistical analysis. All values were shown as means ± SEM of at least three independent experiments. For the analysis of differences between groups, independent-samples t test or one-way ANOVA was performed, and differences were considered significant for P < 0.05.

## Result

### FoxM1 is dynamically expressed in human menstrual endometrium

**Figure 1:**
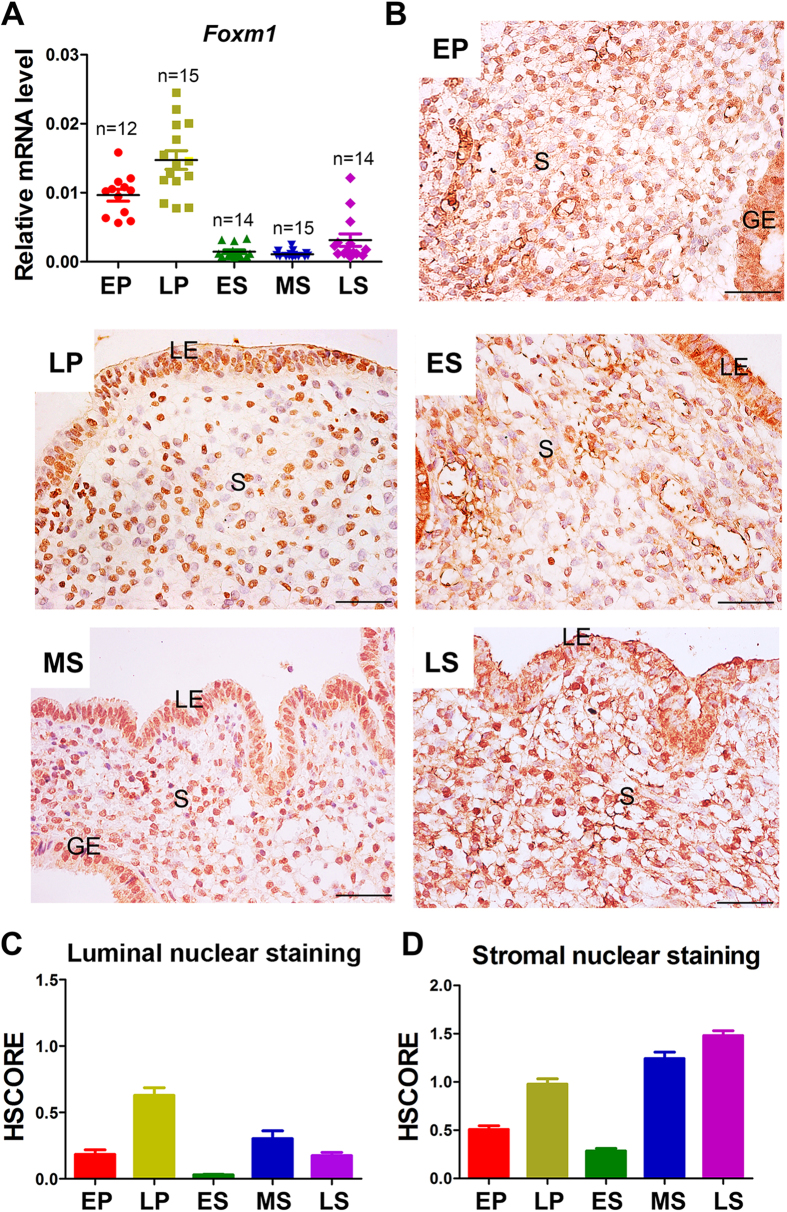
FoxM1 is dynamically expressed in human endometrium at different stages across menstrual cycle. (**A**) FoxM1 mRNA level in human endometrium. The values are normalized to the GAPDH expression level and indicated as the mean ± SEM. LP vs other groups: p < 0.05. (**B**) Immunohistochemical staining for FoxM1 in human endometrium. Scale bars represent 50 μm. (**C**,**D**) HSCORE of nuclear immunostaining for FoxM1 in epithelium (**C**) and stroma (**D**). Data shown represent the mean ± SEM. n = 10. EP, early proliferative phase; LP, late proliferative phase; ES, Early secretory phase; MS, Mid-secretory phase; LP, Late secretory phase; S, stromal cells; GE, glandular epithelium; LE, luminal epithelium. HSCORES analysis reveals a significant increase in nuclear stromal FoxM1 expression in the LP, MS and LS phases (p < 0.05).

To explore the pathophysiological significance of FoxM1 in human endometrial function, we collected human endometrial biopsies in different phase of menstrual cycle from 70 normally cycling volunteers. The expression of FoxM1 mRNA and protein in endometrial tissues was detected by q-PCR and immunohistochemistry, respectively. The mRNA expression for FoxM1 was different across the menstrual cycle with higher expression in the proliferative phase but lower expression in the early-secretory and mid-secretory phase. Interestingly, there was a dramatic increase in the late-secretory phase (Fig. 1A). With regard to the expression localization, FoxM1 was expressed in both cytoplasm and nuclei in the luminal and glandular epithelium as well as the stromal cells not only in the proliferative phase, but also in the secretory phase (Fig. 1B). Since FoxM1 acted specifically in the nucleus, we conducted specific assessment of nuclear staining by HSCORE analysis in both epithelial and stromal compartments. As shown in Fig. 1C, the nuclear staining of FoxM1 in epithelial compartment reveled apparent increase in late proliferative phase in comparison with other phases. In the stromal compartment, the HSOCRE of stromal nuclear staining showed significant increases in not only the late proliferative phase but also the mid-secretory phase (Fig. 1D). Overall, these results suggested that FoxM1 might have a key role in the cyclic endometrial proliferation and differentiation. In this study we mainly focus on exploring the potential roles of FoxM1 in stromal cells, since its timely nuclear accumulation in stroma at mid-secretory phase when the endometrium enters the receptivity status and undergoes stromal-decidual transformation.

### FoxM1 is required for human endometrial stromal cell proliferation, whereas its inhibition induces cell proliferation arrest at the G2/M phase.

**Figure 2:**
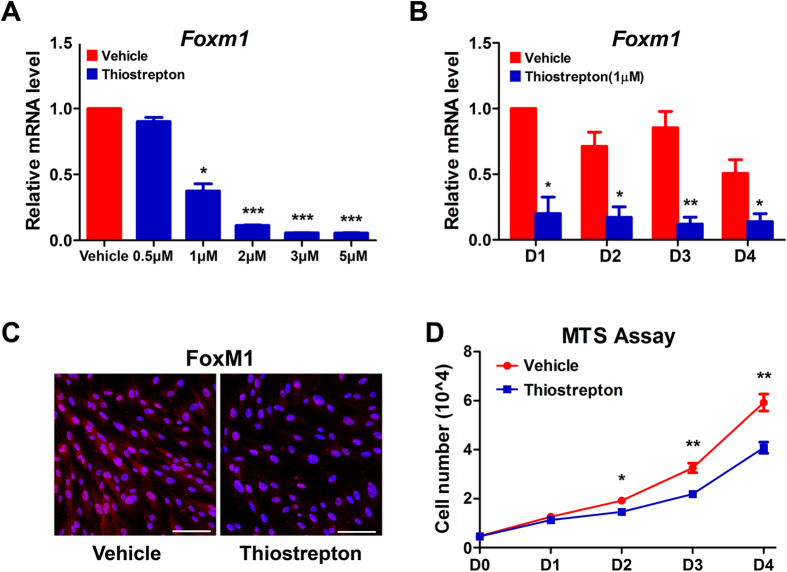
FoxM1 is required for human endometrial stromal cell proliferation. (**A**) FoxM1 mRNA level after treatment with 0 μM, 0.5 μM, 1 μM, 2 μM, 3 μM or 5 μM of thiostrepton. The values are normalized to the GAPDH expression level and indicated as the mean ± SEM. n = 3. *P < 0.05, ***P < 0.001. (**B**) FoxM1 mRNA level after treatment with 1 μM thiostrepton at days 1–4. The values are normalized to the GAPDH expression level and indicated as the mean ± SEM. n = 3. *P < 0.05, **P < 0.01. (**C**) Immunocytochemistry staining of FoxM1 in HESCs treated with vehicle or 1 μM thiostrepton for 2 days. Scale bars represent 100 μm. (**D**) Measurements of cell proliferation by MTS assay after treatment with 1μmthiostrepton at days 0–4. The values represent the mean ± SEM of six replicates from three independent experiments. *P < 0.05, **P < 0.01.

To study the role of FoxM1 during human endometrial stromal proliferation and decidualization, thiazole antibiotic thiostrepton, a pharmacological FoxM1 inhibitor that has previously been showed to inhibit the FoxM1 expression and its transcriptional activity^25^,^26^ was used in HESCs in culture. To find the appropriate concentration of thiostrepton in FoxM1 functional inhibition, we treated HESCs with various doses of thiostrepton from 0 to 5 μM. As shown in Fig. 2A, FoxM1 mRNA expression was largely inhibited with increasing doses of thiostrepton, exhibiting a significant FoxM1 reduction at a concentration of 1 μM without signs of massive cell death (Supplemental Figure 1A–F). Therefore, 1 μM of thiostrepton was used in subsequent time-course experiments. As shown in Fig. 2B, 80% reduction of FoxM1 mRNA expression was noted in cultured HESCs on days 1–4 after thiostrepton treatment. Immunocytochemistry analysis revealed a similar reduced FoxM1 protein expression following treatment with thiostrepton (Fig. 2C). MTS assay was then performed to determine the HESC proliferation status upon FoxM1 inhibition. As shown in Fig. 2D, HESC proliferation activity in thiostrepton-treated group was significantly attenuated compared with the control.

**Figure 3:**
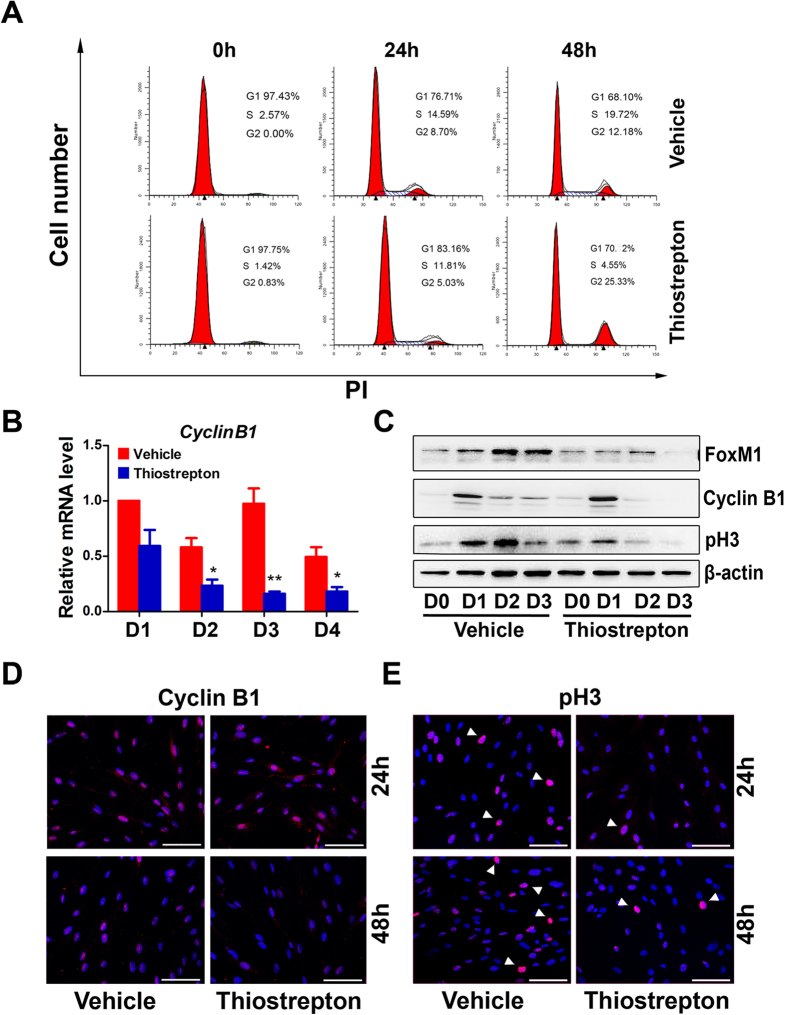
FoxM1 inhibition induces cell proliferation arrest at the G2/M phase. (**A**) FACS analysis of HESCs treated with vehicle or 1 μmthiostrepton at 0 h, 24 h and 48 h. The percentage of cells at the G1, S and G2/M phase was indicated in figures. (**B**) Cyclin B1 mRNA level in HESCs treated with vehicle or 1 μm thiostrepton at days 1–4. The values are normalized to the GAPDH expression level and indicated as the mean ± SEM. n = 3. *P < 0.05, **P < 0.01. (**C**) Western blot analysis of FoxM1, cyclin B1 and pH3 in HESCs treated with vehicle or 1 μm thiostrepton at days 0–3. (**D**,**E**) The immunocytochemistry staining of cyclin B1 (**D**) and pH3 (**E**) in HESCs treated with vehicle or 1 μm thiostrepton at 24 h and 48 h, respectively. The white arrowheads denote pH3 expressing HESCs. Scale bars represent 100 μm.

A wealth of evidence supported a role of FoxM1 in modulating cell cycle progression including G1/S and G2/M transition^15^. To clarify the cell cycle status upon FoxM1 inhibition during HESC proliferation, we performed flow cytometric analysis. HESCs were serum starved to synchronize the cell cycle at the G0/G1 stage, which then were released by culture in medium containing serum. As shown in Fig. 3A, while vehicle-treated HESCs normally went through each stages of cell cycle from the initial G0/G1 into the S and later through the G2/M phase at approximate every 24 h, a significantly increased ratio of HESCs were arrested in the G2/M phase at 48 h post thiostrepton treatment. These results indicated that the downregulation of FoxM1 derails the entry and progression of HESCs into the M phase.

Since cyclin B1 was well-known for its necessity during the G2/M transition^27^, we then investigated the mRNA expression of this checkpoint molecule in proliferating HESCs in response to FoxM1 inhibition. As expected, cyclin B1 mRNA expression, which reached a peak at 3 days after the termination of serum starvation in control group, was significantly reduced in HESCs treated with thiostrepton (Fig. 3B). A drastic reduction of cyclin B1 protein expression was also accordingly noted following thiostrepton-inhibited FoxM1 expression and activation (Fig. 3C,D and Supplemental Figure 2A and B). To further confirm the G2/M transition defect in HESC proliferation in response to FoxM1 inhibition, the cellular mitotic activity was examined using an antibody that recognized the unique mitotic phase marker phosphorylated Ser 10 of histone 3 (pH3). In consistent with the impaired cyclin B1 expression, pH3-immunopositive HESCs and pH3 expression levels were remarkably reduced when treated with thiostrepton (Fig. 3C,E, and Supplemental Figure 2C), reinforcing the notion of G2/M arrest resulted from FoxM1 inhibition. However, this derailed cell cycle progression and restrained cell proliferation by FoxM1 inhibition exhibited no apparently adverse effects on cell viability, since we observed comparable expression levels of apoptosis-associated proteins including cleaved Caspase 3, Caspase 3 and Caspase 9, as well as cleaved PARP in vehicle versus thiostrepton-treated HESCs (Supplemental Figure 3A–E). Collectively, these data suggested that inhibition of FoxM1 expression and activation by thiostrepton leads to impaired cell cycle progression rather than apoptosis in HESCs.

### FoxM1 via directly targeting STAT3 ensures human endometrial stromal differentiation and decidualization

**Figure 4:**
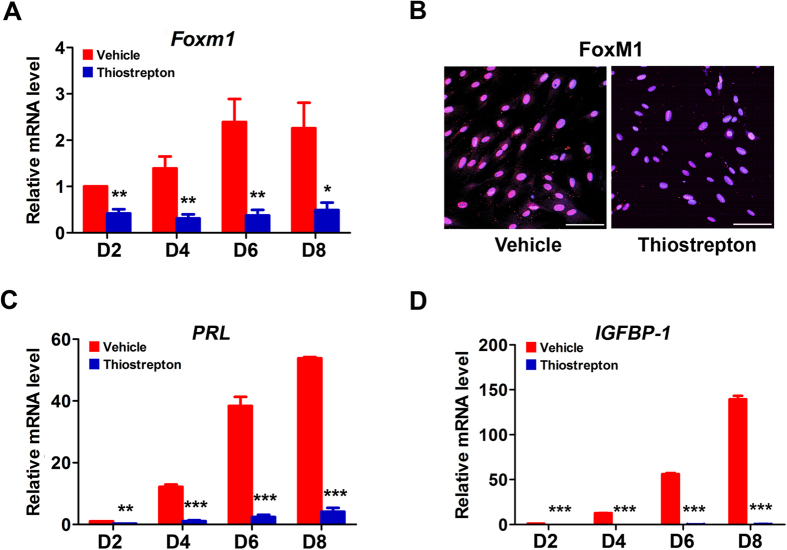
FoxM1 regulates E_2_, MPA and dbcAMP-induced HESC differentiation in culture. (**A**) FoxM1 mRNA level in HESCs after treatment with 1 μM thiostrepton at days 2–8. The values are normalized to the GAPDH expression level and indicated as the mean ± SEM. n = 3. *P < 0.05, **P < 0.01. (**B**) Immunocytochemistry staining of FoxM1 in HESCs treated with vehicle or 1 μM thiostrepton for 8 days. Scale bars represent 100 μm. (**C**,**D**), Relative mRNA expression of PRL (**C**) and IGFBP-1 (**D**) in HESCs treated with vehicle or 1 μm thiostrepton at days 2–8. The values are normalized to the GAPDH expression level and indicated as the mean ± SEM. n = 3. **P < 0.01, ***P < 0.001.

Observations of strong nuclei localization of FoxM1 protein during mid- and late secretory stage (Fig. 1B,C) inspired us to investigate its potential function during stromal differentiation. As previously described^22^, HESCs in culture can undergo extensive stromal-decidual transformation in response to E_2_, MPA and dbcAMP. While treatment of thiostrepton significantly reduced both FoxM1 mRNA and protein expressions in HESCs (Fig. 4A,B), this FoxM1 inhibition remarkably attenuated the mRNA expressions of PRL and IGFBP-1 (Fig. 4C,D), indicating an impaired decidualization upon FoxM1 inhibition. These results revealed an essential role of FoxM1 during endometrial stromal differentiation.

**Figure 5:**
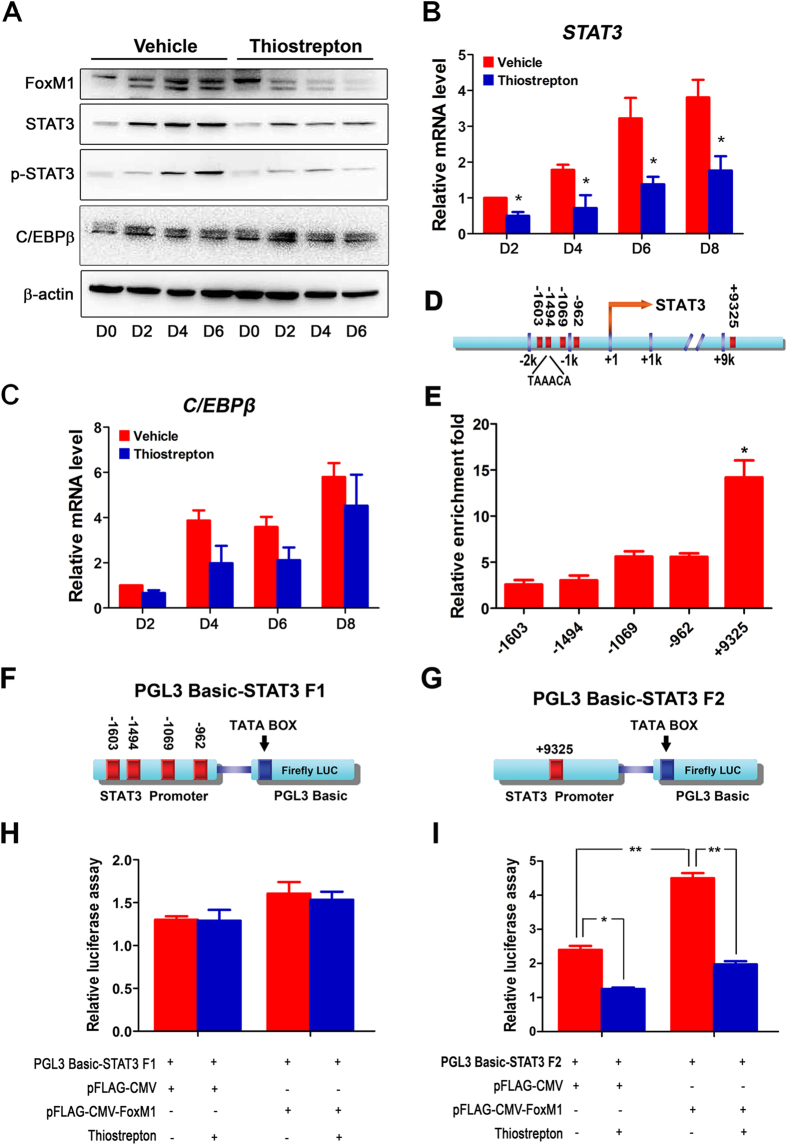
FoxM1 directly targets STAT3 in HESCs during E_2_, MPA and dbcAMP-induced differentiation. (**A**) Western blot analysis of FoxM1, STAT3, p-STAT3 and C/EBPβ in differential HESCs treated with vehicle or 1 μm thiostrepton at days 0, 2, 4, 6, respectively. (**B**,**C**) Relative mRNA expression of STAT3 (**B**) and C/EBPβ (**C**) in HESCs treated with vehicle or 1 μM thiostrepton at days 2, 4, 6 and 8. The values are normalized to the GAPDH expression level and indicated as the mean ± SEM. n = 3. *P < 0.05. (**D**) Schematic representation of STAT3 promoter region with potential FoxM1 binding sites indicated by red bands. (**E**) The relative enrichment fold of different FoxM1 binding sites compared with IgG was expressed by ChIP-qPCR. The values represent the mean ± SEM of six replicates from three independent experiments. *P < 0.05. (**F**,**G**) Schematic diagram of the PGL3-STAT3 F1 (**F**) and PGL3-STAT3 F2 (**G**) reporter constructs for the promoter assay. (**H**,**I**) Luciferase assays of *Cis*-activation potential of the region containing PGL3-STAT3 F1 (**F**) and PGL3-STAT3 F2 (**G**) in the presence of the control plasmid, FoxM1 over-expression plasmid or 1 μM thiostrepton. The values are shown as the mean ± SEM. n = 3. *P < 0.05, **P < 0.01.

Previous study evidenced that transcriptional C/EBPβ-STAT3 cascades involves in human endometrial decidualization^13^. To reveal the underlying mechanism by which FoxM1 governs the normal process of human endometrial stromal differentiation, we first examined the expression of STAT3 and C/EBPβ after FoxM1 inhibition by thiostrepton in the differentiating HESCs, As shown in Fig. 5A,B and indexed in Supplemental Figure 4A and B, we observed that FoxM1 inhibition in the differentiating HESCs led to a significant reduction of both STAT3 mRNA and protein expression. Moreover, the phosphorylation activation of STAT3 was also accordingly attenuated in HESCs upon FoxM1 inhibition (Fig. 5A, Supplemental Figure 4C). Interestingly, STAT3 inhibition by its selective inhibitor pyridone 6 also resulted in an impaired HESC decidualization in culture (Supplemental Figure 5A). These data provided the possibility regarding the potential interactions between FoxM1 and STAT3 signaling. Although STAT3 is a downstream effector of C/EBPβ during endometrial decidualization^13^, we found C/EBPβ was apparently normally expressed even after FoxM1 inhibition (Fig. 5A,C, Supplemental Figure 4D). These results indicated that FoxM1 involves in HESC decidualization most likely through regulating the STAT3 expression.

To further verify whether STAT3 is a direct targeting gene of FoxM1 transcription activation, we performed ChIP-qPCR experiments. Bioinformatic analysis of the STAT3 promoter revealed that it contains five candidate FoxM1 binding motifs (TAAACA), displaying four of them within the −2 kb 5′-flanking region of the gene body at nucleotides −962, −1069, −1494 and −1603 relative to the transcription start site, and another one at nucleotides +9325 relative to the transcription start site (Fig. 5D). As shown in Fig. 5E, by ChIP-qPCR analysis, we observed minimal binding of FoxM1 at the respective binding motifs at −962, −1069, −1494 and −1603 of STAT3 promoter, whereas a relatively strong enriched FoxM1 binding at the +9325 region. To confirm that the TAAACA motif at +9325 is indeed the site by which endogenous FoxM1 regulates the STAT3 expression, we performed a dual-luciferase assay using reporter constructs containing STAT3 F1 (−755 ∼ −1834) or STAT3 F2 (+9325 ∼ +9580) on HEK 293T cell line, respectively (Fig. 5F,G). While either gain or loss of FoxM1 function exhibited no influence on STAT3 F1 luciferase activity (Fig. 5H), a co-transfection of STAT3 F2 with FoxM1 significantly enhanced luciferase activity, and FoxM1 inhibition by thiostrepton efficiently reversed this stimulating effect (Fig. 5I). This finding confirmed our contention that STAT3 is a direct target gene of FoxM1 transcription regulating human endometrial stromal cell function.

## Discussion

Endometrium is an active participant and key determinant of successful embryo implantation^3^. There is emerging concept showing that decidualized endometrium could serve as a sensor of embryo quality^28^. For example, embryo signaling can direct the functional alteration of the endoplasmic reticulum within decidualized HESCs that undergo a stress or nurturing response once the embryo breaches the luminal epithelium^29^. Moreover, HESCs have been shown to be motile, engaging actively in either supporting the embedding of the embryo, or indeed acting to discourage this^30^. In the present study, we demonstrated that the transcriptional factor FoxM1 is indispensable for normal endometrial stromal cell cycle progression, and thus cell proliferation, while FoxM1 by directly targeting STAT3 transcription ensures normal stromal cell differentiation and decidualization in human endometrial cells in culture.

In our study, we obtained total 70 human endometrium samples to study the dynamic FoxM1 expression during menstrual cycle. Our mRNA expression analysis results showed a peak FoxM1 mRNA expression in the proliferative phase and late secretory phase in whole samples of human endometrium, suggesting a potential role of FoxM1 in preparation of human menstruation. Although we did not found an apparent FoxM1 mRNA increase in the mid-secretory phase (the human stromal decidualization begins during the phase)^2^, we noticed with interests by immunohistochemistry staining analysis that nuclear FoxM1 was increased markedly in the subepithelial stromal cells in the mid-secretory phase, which coincides with the opening of the window of receptivity to embryo implantation. Besides the higher expression in the endometrial stroma, we also found a strong staining of FoxM1 in the epithelial compartment. Therefore, it will be interestingly to further elucidate its role in the epithelium, especially the potential functions under different hormone treatment in future.

Although recent studies employing mouse models have indicated uterine FoxM1 expression is under a profound influence of ovarian steroid hormones^31^, our observations of dynamic FoxM1 expression with a peak mRNA level in the proliferative phase and late secretory phase in human endometrium add new line of evidence emphasizing its potential role during endometrial stromal proliferation and differentiation for successful implantation in humans. Moreover, we proved that FoxM1 is essential for stromal cell proliferation rather than cell viability, via ensuring normal cell cycle G2/M transition and progression. This is consistent with previous observations that loss of FoxM1 results in pleiotropic cell cycle defects, including a delay in G2, chromosome mis-segregation and frequent failure of cytokinesis^32^. It has been well documented that FoxM1 plays a key role in regulating specific cell cycle related gene expressions^15^. In this respect, we demonstrated that FoxM1-targeting inhibition and downregulation can remarkably attenuate the cyclin B1 expression, which in combination with CDK1 is known required for G2/M transition^27^,^33^. In fact, there is evidence that cyclin B1 could be transcriptionally regulated by FoxM1 to mediate timely mitotic entry^32^. It is conceivable that FoxM1-cyclin B1 cascade could be a common machinery governing cell proliferation in various pathophysiological events and cell-types, including in human endometrium.

As above-stated, the endometrium will undergo dynamic remodeling under the influence of steroid hormones. The postovulatory increase of progesterone and putative factors from the implanting blastocyst, elevates intercellular cAMP, thereby activating the protein kinase A signaling pathway that triggers full stromal differentiation^34^. Besides the most principle transcriptional regulatory circuit driven by estrogen and progesterone and their respective nuclear receptors, it remained to uncover the new players contributing the regulatory circumstance. Moreover, it would be of great interests to see how these transcription regulators interacting with other signaling cascades. In this respect, we herein provided novel evidence that FoxM1 is not only essential for normal human endometrial stromal cell proliferation, but most interestingly, it can also directly regulate the transcriptional activation of STAT3 ensuring normal stromal decidualization. JAKs-STAT3 pathway is a common signaling cascade taking part in human endometrial decidualization^3^,^35^,^36^. Previous studies have demonstrated that STAT3 can function as a direct regulatory target of PR-C/EBPβ involved in human endometrial decidualization^11^,^12^,^13^. However, FoxM1 inhibition failed to exert any adverse effects on C/EBPβ expression, suggesting potential diversified regulatory machineries on STAT3 expression during endometrial stromal differentiation. Interestingly, there is evidence that FoxM1 is an intermediate upon STAT3 phosphorylation activation in proliferation, survival and DNA repair of chronic myeloid leukemia^37^. In addition, a most recent report showed that FoxM1 drives a feed-forward STAT3-activation signaling loop involved in promoting the self-renewal and tumorigenicity of glioblastoma stem-like cells^38^. However, we failed to note this back-forward feedback loop between FoxM1 and STAT3 during endometrial decidualization, since we observed that blockage of STAT3 activation has no influence on FoxM1 expression (unpublished data), although this inhibition can largely hamper the decidualization process.

In summary, we provide here multiple lines of evidence demonstrating a unique phenomenon in endometrium: while FoxM1 is essential for cell cycle progression and thus cell proliferation via maintaining dynamic cyclin B1 expression, it functions through STAT3 signaling ensuring normal endometrial decidualization. Besides adding new knowledge to better understand stromal decidualization, our findings have clinical relevance since aberrant decidualization of HESCs is closely associated with recurrent pregnancy loss.

## Additional Information

**How to cite this article**: Jiang, Y. *et al*. FoxM1 Directs STAT3 Expression Essential for Human Endometrial Stromal Decidualization. *Sci. Rep*. **5**, 13735; doi: 10.1038/srep13735 (2015).

## Supplementary Material

Supplementary Information
