# Supplementary material for: FoxM1 Directs STAT3 Expression Essential for Human Endometrial Stromal Decidualization
Source: Sci Rep. 2015 Sep 3;5:13735. doi: 10.1038/srep13735 (PMC4558550; doi:10.1038/srep13735)
Supplement: Supplementary Information [file srep13735-s1.pdf]

FoxM1 Directs STAT3 Expression Essential for Human Endometrial Stromal Decidualization

Yaling Jiang, Yixin Liao, Hui He, Qiliang Xin, Zhaowei Tu, Shuangbo Kong, Tongtong Cui, Bingyan Wang, Song Quan, Bing Li, Shuang Zhang, Haibin Wang

Supplemental Tables:

Supplemental Table 1. Primer sequences of target genes.

Gene	Forward primer	Reverse primer	Length (bps)
q-PCR primer			
FoxM1	GACATCAGCTTTCCTGGCCT	CAGTGGCTAGGGTGTGACTG	248
CyclinB1	TCTGGATAATGGTGAATGGA	GATGTGGCATACTTGTTCTT	156
PRL	CTACATCCATAACCTCTCCTCAG	GGGCTTGCTCCTTGTCTTC	137
IGFBP-1	AGAGTCGTAGAGAGTTTAGC	ACACTGTCTGCTGTGATAA	108
STAT3	CTCCACTGGTCTATCTCTATC	ACTTGGTCTTCAGGTATGG	126
C/EBPβ	AACTCTCTGCTTCTCCCTCTG	TGCGTCAGTCCCGTGTAC	113
GAPDH	ATTTGGCTACAGCAACAGG	TTGAGCACAGGGTACTTTATT	256
ChIP q-PCR primer			
STAT3（-962）	TTTGTCTTTATACCTGTTCGTATT	GCATAATGGAGTGATTTCTGT	126
STAT3（-1069）	TCAAAGGTTAAGAGTCATTGATT	CATGTTCCTGTTTAGAAAATGC	147
STAT3（-1494）	ATAGCGTAGTTACAAGAGACT	TGGTGGTGAGAATTGAGAA	165
STAT3（-1603）	CCTCTCCTATCTGCTATTCAT	AGTCTCTTGTAACTACGCTAT	159
STAT3（+9325）	AACCTTCTGGGAAATAAACG	CCTAAATTGGGCTTACCTTC	137
Luciferase reporter cloning primer			
STAT3 F1(-755~ -1834)	GCAGGGTTTGAATGAATGGAACA	TTTCTTTTCCACCTTGCCTACT	1080
STAT3 F2(+9325~+9580)	AGGAAGAAGCCAGCAGAGCTTGA	TGAGAGGTCAGAATACCACAA	255

Supplemental Table 2. Baseline characteristics of RIF patients and the controls.

	RIF (n=15)	Control (n=15)	P value
Age	29.93±1.87	30.27±1.83	0.63
Endometrium thickness (mm)	8.58±1.58	9.13±1.81	0.39
FSH (mIU/ml)	2.04±0.23	1.99±0.22	0.59
LH (mIU/ml)	2.08±0.22	2.03±0.37	0.70
E2 (pg/ml)	300.32±24.63	296.69±12.99	0.62
P4 (ng/ml)	22.44±1.91	22.19±2.00	0.73

Data are presented as mean ± SD. FSH, follicles timulating hormone; LH, luteinizing hormone; E2, oestradiol; P4, progesterone.

Supplemental Figures

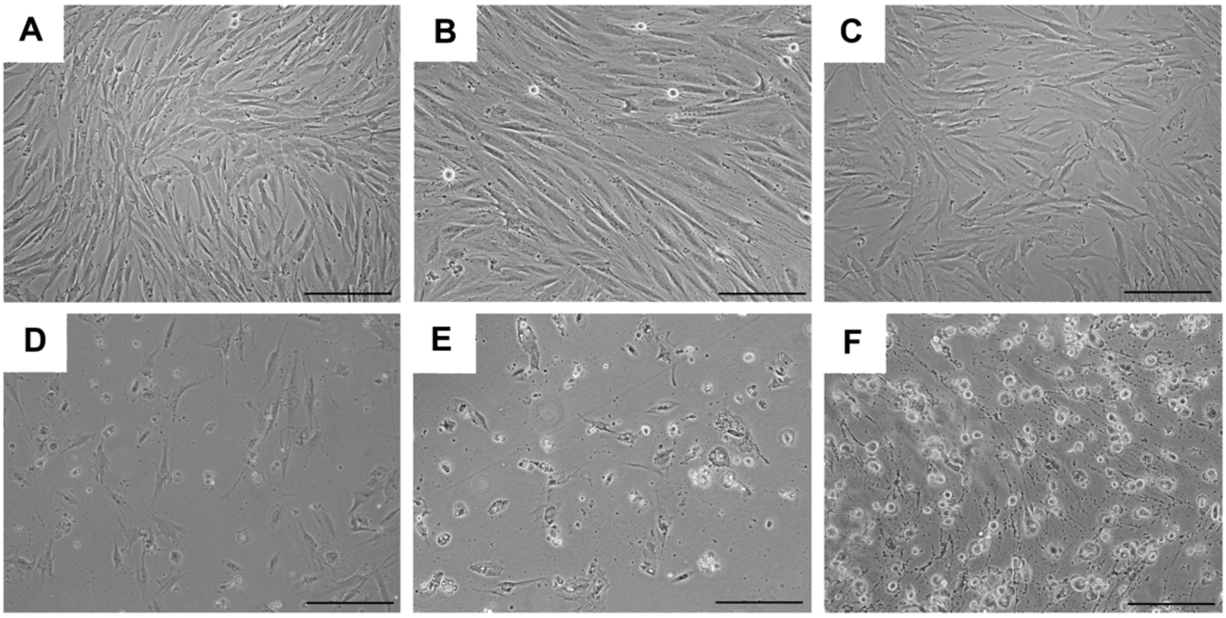

Supplemental Fig. 1 Representative HESCs cultured in proliferative medium treated with vehicle orvarious doses of thiostrepton for 2 days. A, vehicle; B, 0.5 μM; C, 1μM; D, 2 μM; E, 3 μM; F, 5 μM.

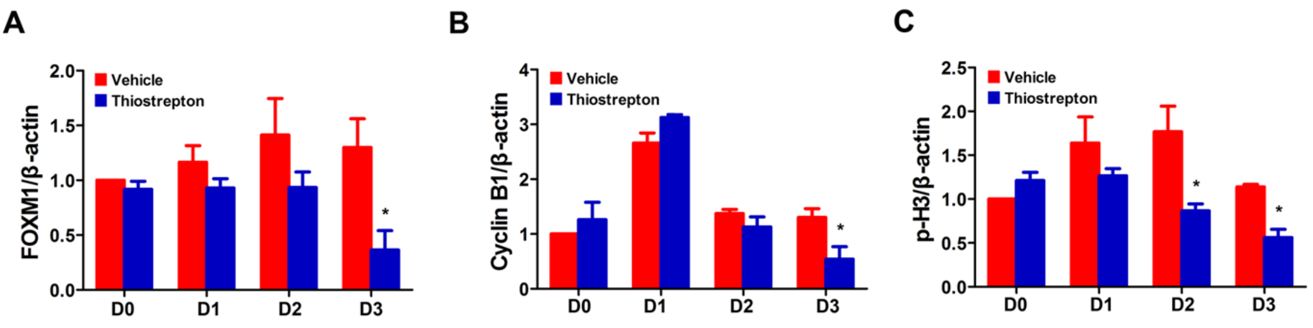

Supplemental Fig. 2 Gray-scales canning of immunoblot bands corresponding to FoxM1, cyclinB1 and pH3 in Figure 3C. Datashown represent the mean ± SEM from three independent experiments. *P < 0.05.

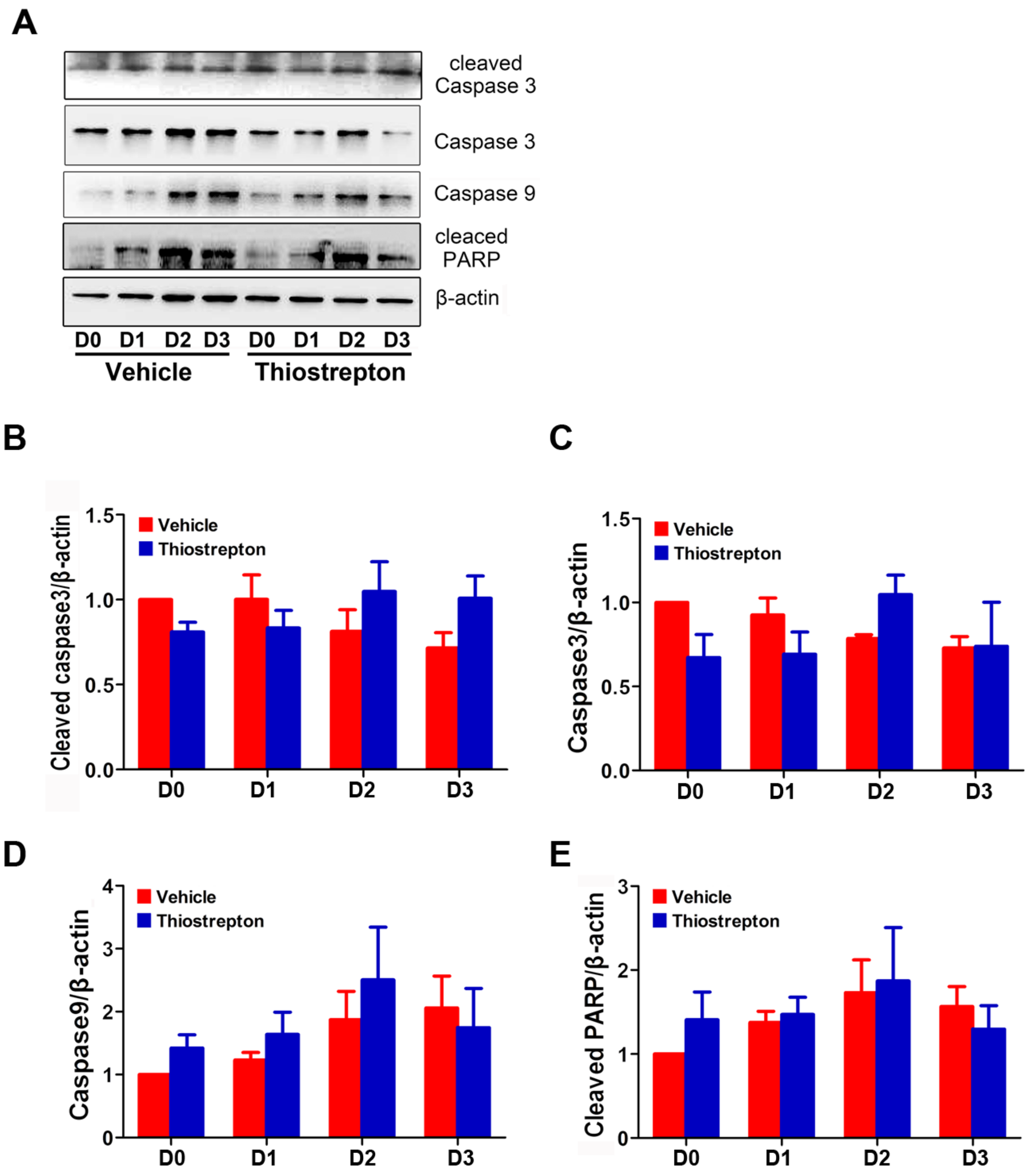

Supplemental Fig. 3 Western Blot (A) and relative gray-scale scanning analysis of cleaved Caspase3 (B), Caspase3 (C), Caspase9 (D) and cleaved PARP (E) in HESCs treated with vehicle or 1 μM thiostrepton at days 0-3. Data shown represent the mean ± SEM from three independent experiments. *P < 0.05.

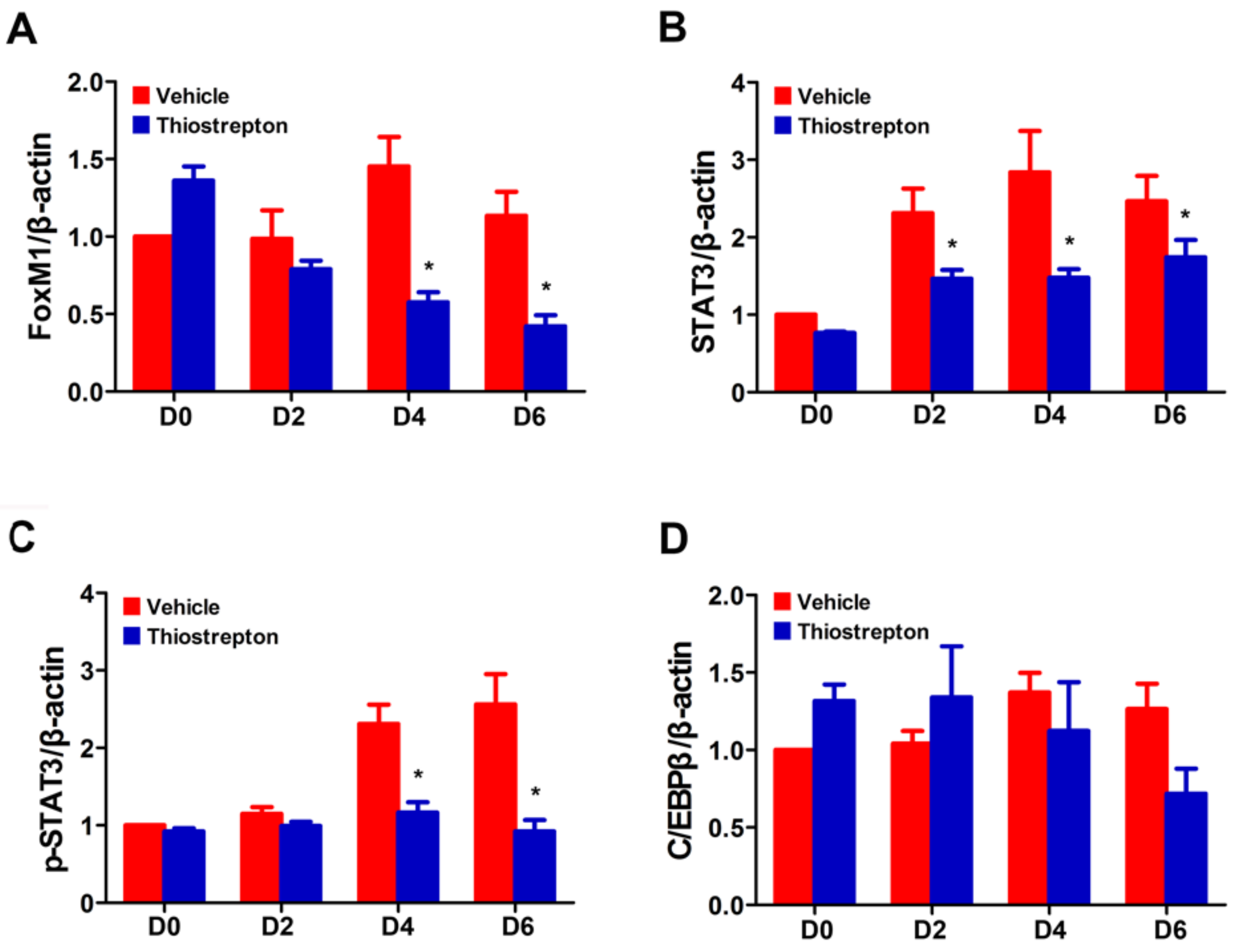

Supplemental Fig. 4 Gray-scalescanning of immunoblot bands corresponding to FoxM1 (A), STAT3 (B), p-STAT3 (C) and C/EBPβ (D) in Figure 5A. Data shown represent the mean ± SEM from three independent experiments.

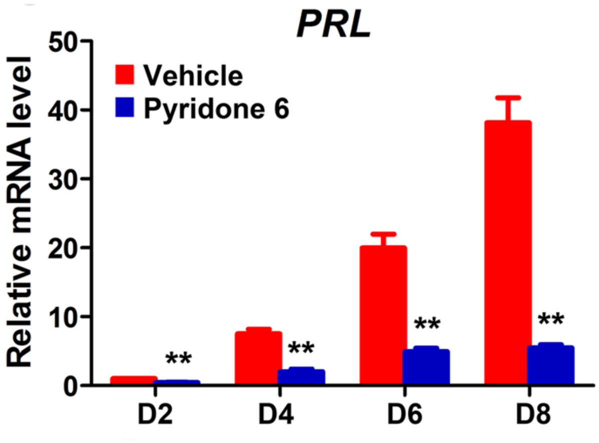

Supplemental Fig. 5 PRL mRNA level in differential HESCs treated with vehicle or 1 μM pyridone 6 at days 2, 4, 6 and 8. The values are normalized to the GAPDH expression level and indicated as the mean ± SEM. n=3.**P < 0.01.
